# Evaluation of ophthalmic healthcare professional-led keratoconus management service in the United Kingdom: the Birmingham and Midland Eye Centre (BMEC) study

**DOI:** 10.1038/s41433-024-03169-z

**Published:** 2024-06-13

**Authors:** Marianthi Bourlaki, Murad Khan, Saliamma Bandyopadhyay, Rashvinder Sahota, Emadur Khan, Urvasee Patel, Mykolas Pajaujis, Anil Aralikatti, Ankur Barua, Darren S. J. Ting

**Affiliations:** 1 Birmingham and Midland Eye Centre, Sandwell and West Birmingham Hospitals NHS Trust, Birmingham, UK; 2 Academic Unit of Ophthalmology, Institute of Inflammation and Ageing, University of Birmingham, Birmingham, UK; 3 Academic Ophthalmology, School of Medicine, University of Nottingham, Nottingham, UK

**Keywords:** Health services, Health occupations, Corneal diseases

Keratoconus is the most common corneal ectatic disorder, affecting 1:375 to 1:2000 people globally [1]. Timely diagnosis and early intervention such as corneal cross-linking (CXL) is essential to stabilise progressive keratoconus, preserve vision and reduce the need for corneal transplantation [2]. In view of improved community screening [3], ophthalmic units across the UK are seeing a significant increase in community-to-hospital referrals for keratoconus management. To cope with the increased workload, several innovative pathways/services have been established, including the setup of virtual keratoconus clinics and CXL services led by nurses and optometrists [collectively known as ophthalmic healthcare professionals (OHPs)] [4, 5]. This cross-sectional study aimed to examine the effectiveness and safety of OHP-led keratoconus service in Birmingham and Midland Eye Centre (BMEC), one of the largest ophthalmic tertiary referral centres in the UK.

This study was approved by the clinical governance team of the Sandwell and West Birmingham Hospitals NHS Trust as a clinical audit (Ref: 2411). We included 170 patients with suspected/confirmed keratoconus from OHP-led keratoconus clinics at BMEC, run by two nurses (SB, RS) and two optometrists (EK, UP) with 3–9 years of corneal experience, between June 2023 and October 2023. Relevant data, including demographic factors, previous ocular/medical history, use of CL, corrected-distance-visual-acuity (CDVA), corneal tomographic findings, clinical diagnosis, and management, were collected from the local electronic patient record and Pentacam machine (Table 1). Progression of keratoconus was defined by the presence of ≥2 following parameters over the past year (adapted from previous studies) [1, 2]:

- Subjective/objective decrease in CDVA ≥ 1 Snellen line
- ≥1D increase in cylinder on manifest refraction
- ≥1D increase in K2
- ≥1D increase in Kmax
- Progressive thinning of corneal thickness

**Table 1:** Summary of the baseline characteristics of patients with suspected keratoconus (KC) in Birmingham and Midland Eye Centre, United Kingdom.

Parameters	*N* (%)
Age, years	30.9 (10.6)
Gender	
Female	69 (40.6)
Male	101 (59.4)
Nature of Visit	
New	53 (31.2)
Follow-up	117 (68.8)
Diagnosis	
Bilateral KC	142 (83.5)
Unilateral KC + normal fellow eye	23 (13.5)
Unilateral KC + suspected KC in fellow eye	4 (2.4)
Bilateral suspected KC	1 (0.6)
Presenting CDVA, logMAR	
Right eye	
≥0.3	130 (76.5)
<0.3 to 0.6	27 (15.9)
<0.6 to 1.0	10 (5.9)
<1.0	3 (1.8)
Left eye	
≥0.3	123 (72.4)
<0.3 to 0.6	32 (18.8)
0.6 to 1.0	5 (2.9)
<1.0	10 (5.9)
K1 (Flat keratometry), D	
Right eye	46.3 (5.5)
Left eye	51.0 (5.0)
K2 (Steep keratometry), D	
Right eye	49.5 (6.9)
Left eye	50.3 (8.1)
Kmax, D	
Right eye	53.3 (9.6)
Left eye	54.8 (11.3)
Thinnest pachymetry, microns	
Right eye	465.9 (53.8)
Left eye	457.5 (60.8)

*CDVA* Corrected-distance-visual-acuity.

The decision was first made by OHPs independently and was recorded as either observe, list for CXL, discharge, or uncertain. This was then compared to the decision made independently by the corneal consultant ophthalmologists. The main outcome measure was the concordance of the clinical decision made between OHPs and corneal consultants.

53 (31.2%) and 117 (68.8%) were of new and follow-up visits, respectively. OHPs made decisions to observe in 141 (82.9%), list for CXL in 11 (6.5%) cases, discharge in 8 (4.7%) cases, and expressed uncertainty in only 10 (5.9%) cases. An excellent agreement (91.8%) was observed between OHPs and consultants (Table 2). Of the 10 (5.9%) cases with uncertainty, the consultant recommended observation in 5 cases, CXL in 4 cases and discharge in 1 case. For the four remaining cases with disagreement, OHPs made the decision to observe but the consultant made the decision to discharge. Six cases were correctly listed for CXL at the first visit, and no cases were incorrectly listed for CXL or discharge by the OHPs.

**Table 2:** Concordance of the clinical decision made for the management of keratoconus between the ophthalmic healthcare professionals (OHPs) and the cornea consultants in Birmingham and Midland Eye Centre, UK.

		Ophthalmic healthcare professionals			
		*Observe*	*CXL*	*Discharge*	*Uncertain*
Cornea consultants	*Observe*	137 (80.6%)	0 (0.0%)	0 (0.0%)	5 (2.9%)
		(43N, 94F)	–	–	(2N, 3F)
	*CXL*	0 (0.0%)	11 (6.5%)	0 (0.0%)	4 (2.4%)
		–	(6N, 5F)	–	(1N, 3F)
	*Discharge*	4 (2.6%)	0 (0.0%)	8 (4.7%)	1 (0.6%)
		(0N, 4F)	–	(0N, 8F)	(1N, 0F)

Shaded areas refer to clinical decisions that were made in agreement between OHPs and cornea consultants.

*CXL* Corneal cross-linking, *N* New patients, *F* Follow-up patients.

This study highlights an excellent concordance in clinical decision-making between OHPs and corneal consultant ophthalmologists for managing new and follow-up cases of suspected keratoconus, highlighting the effectiveness, safety and feasibility of OHP-led keratoconus services (with minimal consultant support). Currently, various definitions of keratoconus progression exist in the literature and clinical practice, including the Belin ABCD Progression Display [1, 6]. In 2015, the Global Delphi Panel of Keratoconus and Ectatic Disease defined ectasia/keratoconus progression by a consistent change in ≥2 of the following parameters, including progressive steepening of the anterior or posterior corneal surface or progressive corneal thinning, though no specific quantitative threshold/value was provided [7]. Bearing these issues in mind, our study showed that, with a standardised and relatively simplified protocol, an effective and safe OHP-led keratoconus service model can be set up, and similar models can potentially be replicated in other UK ophthalmic units to help alleviate the pressure of current ophthalmic services. Furthermore, with the recent advancement in deep learning technology (a subset of artificial intelligence) and availability of big data, it is envisaged that artificial intelligence will likely play an important and valuable role in assisting the clinical management of keratoconus and other corneal diseases in the future [8, 9].
