# Correction: Evaluation of ophthalmic healthcare professional-led keratoconus management service in the United Kingdom: the Birmingham and Midland Eye Centre (BMEC) study

**DOI:** 10.1038/s41433-024-03226-7

**Published:** 2024-07-29

**Authors:** Marianthi Bourlaki, Murad Khan, Saliamma Bandyopadhyay, Rashvinder Sahota, Emadur Khan, Urvasee Patel, Mykolas Pajaujis, Anil Aralikatti, Ankur Barua, Darren S. J. Ting

**Affiliations:** 1 Birmingham and Midland Eye Centre, Sandwell and West Birmingham Hospitals NHS Trust, Birmingham, UK; 2 Academic Unit of Ophthalmology, Institute of Inflammation and Ageing, University of Birmingham, Birmingham, UK; 3 Academic Ophthalmology, School of Medicine, University of Nottingham, Nottingham, UK

**Keywords:** Health services, Health occupations, Corneal diseases

Correction to: *Eye* 10.1038/s41433-024-03169-z, published online 13 June 2024

The term “allied health professional(s)” was replaced with “ophthalmic healthcare professional(s)” in the title and throughout the main text of this article. The terminology “allied health professionals” refers to a distinct group of healthcare professionals whereas nurses and optometrists in this context should be referred to as “ophthalmic healthcare professionals”. All research findings and key messages remain the same as in the original version of the manuscript. The original article has been corrected.

